# The Unexpected Hand Patient: Invited Commentary

**Published:** 2017-05-05

**Authors:** Stephen R. Katzman

**Affiliations:** Methfessel & Werbel, PC, Edison, NJ www.methwerblaw.com

The article “The Unexpected Hand Patient” by A. M. Swiergosz, M. L. Kasdan, and B. J. Wilhelmi (*ePlasty.* 2017; 17) demonstrates the clinical presentation of malingering. However, physicians generally treat these patients without an opportunity to experience the full repercussions of this process. It has a huge impact on the business community, the legal system, and the insurance industry. Fraud lawyers expend a considerable effort dealing with the practical reality of malingering. Stephen R. Katzman is an experienced fraud attorney and provided the following information for our readers:

Defense attorneys involved in bodily injury tort litigation (negligence, intentional acts) routinely confront plaintiffs who seek to overtreat and exaggerate symptoms for monetary gain. There was a time when tort lawyers roughly tried to equate the settlement value of a claim for pain and suffering based on 3 times the total of the medical bills. Plaintiff's counsel will encourage the client to seek as much treatment as possible, regardless of whether the treatment is necessary, not only for the stated goal of getting better but also for the unstated goal of enhancing the value of the claim. Typically, if the plaintiff has little to no health care treatment, the claim has little to no value.

Patients who make workers’ compensation claims or federal or state disability claims similarly have a motive to malinger to enhance the value of their claims and/or to avoid work. Other far less prevalent motives for malingering include the desire to obtain prescription medications and the need for attention due to loneliness or a mental disorder. However, it is the monetary incentive to malinger that causes the greatest impact to society because it significantly enhances the cost of insurance premiums and drains government resources.

There is a systemic problem within the auto and slip and fall negligence legal fields when the plaintiff's counsel routinely refers clients to specific health care providers for conveyor belt treatment of alleged soft-tissue injuries. Those health care providers are not interested in malingering and have their own monetary incentives. Clearly, the within commentary is not directed to or being read by those providers.

The honest and ethical health care provider who encounters a potentially malingering patient must make an assessment. Objective testing must be performed, and objective data must be evaluated. There should be a search in the patient records for inconsistencies in patient history and symptom complaints. Is there any objective medical reason for a setback? Reliance on your training and medical literature is of paramount importance. Is the supposed current condition and reversal of direction of recovery inconsistent with what you were taught and what you have read? Does the patient have a motive for exaggeration or fabrication of symptoms, and, if yes, what is it?

If a determination of likely or probable malingering is made, the finding and the basis for the finding should be documented without fear of repercussions. Patients with real medical conditions need to receive timely treatments, not the malingerers. Your valuable time and resources (and the resources of others) should not be wasted. From a legal perspective, the health care provider has every right to set forth his or her opinion and to refuse further services based on the belief that your continued involvement will be of no benefit to the patient. The provider is protected as long as the basis for the finding is clearly set forth in writing. Reliance on the medical literature, including a discussion on malingering in the *Diagnostic and Statistical Manual of Mental Disorders-5* (*DSM-5*), can provide the necessary support for your opinion.

